# The Sick Adipose Tissue: New Insights Into Defective Signaling and Crosstalk With the Myocardium

**DOI:** 10.3389/fendo.2021.735070

**Published:** 2021-09-15

**Authors:** Valmore Bermúdez, Pablo Durán, Edward Rojas, María P. Díaz, José Rivas, Manuel Nava, Maricarmen Chacín, Mayela Cabrera de Bravo, Rubén Carrasquero, Clímaco Cano Ponce, José Luis Górriz, Luis D´Marco

**Affiliations:** ^1^Facultad de Ciencias de la Salud, Universidad Simón Bolívar, Barranquilla, Colombia; ^2^Endocrine and Metabolic Diseases Research Center, School of Medicine, University of Zulia, Maracaibo, Venezuela; ^3^Cardiovascular Division, University Hospital, University of Virginia School of Medicine, Charlottesville, VA, United States; ^4^Department of Medicine, Cardiology Division, University of Florida-College of Medicine, Jacksonville, FL, United States; ^5^City of Houston Health Department, Houston, TX, United States; ^6^Servicio de Nefrología, Hospital Clínico Universitario, INCLIVA, Universidad de Valencia, Valencia, Spain

**Keywords:** adipose tissue, myocardiocytes, microbiota, obesity, inflammation

## Abstract

Adipose tissue (AT) biology is linked to cardiovascular health since obesity is associated with cardiovascular disease (CVD) and positively correlated with excessive visceral fat accumulation. AT signaling to myocardial cells through soluble factors known as adipokines, cardiokines, branched-chain amino acids and small molecules like microRNAs, undoubtedly influence myocardial cells and AT function *via* the endocrine-paracrine mechanisms of action. Unfortunately, abnormal total and visceral adiposity can alter this harmonious signaling network, resulting in tissue hypoxia and monocyte/macrophage adipose infiltration occurring alongside expanded intra-abdominal and epicardial fat depots seen in the human obese phenotype. These processes promote an abnormal adipocyte proteomic reprogramming, whereby these cells become a source of abnormal signals, affecting vascular and myocardial tissues, leading to meta-inflammation, atrial fibrillation, coronary artery disease, heart hypertrophy, heart failure and myocardial infarction. This review first discusses the pathophysiology and consequences of adipose tissue expansion, particularly their association with meta-inflammation and microbiota dysbiosis. We also explore the precise mechanisms involved in metabolic reprogramming in AT that represent plausible causative factors for CVD. Finally, we clarify how lifestyle changes could promote improvement in myocardiocyte function in the context of changes in AT proteomics and a better gut microbiome profile to develop effective, non-pharmacologic approaches to CVD.

## 1 Introduction

Obesity is a chronic and multifactorial metabolic disease described in most scientific literature as the epidemic of the 21st century. In fact, by 2016, this condition affected 650 million adults, equivalent to 13% of the adult population worldwide, while in 2019, 38.3 million children under the age of 5 were overweight or obese (1). In the United States, obesity accounts for approximately 21% of annual national health care costs ($190 billion) (2). In addition, this entity is frequently clustered to other comorbidities such as metabolic syndrome (MetS), insulin resistance (IR), type 2 diabetes mellitus (T2DM), non-alcoholic fatty liver disease (NAFLD), chronic kidney disease (CKD), gout, and cardiovascular disease (CVD) (3). CVD is the leading cause of death worldwide, with approximately 17.9 million deaths each year, of which 85% are attributable to myocardial infarction (MI) and stroke (4).

Research has centered on evaluating the causality of obesity in CVD in recent years, focusing on areas such as the potential role of adipose tissue (AT) on cardiac tissue (5, 6). AT is a highly functional and complex endocrine organ, characterized by the release of adipokines, batokines, microRNAs, prostaglandins, bioactive lipids and other regulators of metabolic homeostasis, which interact with vascular, hepatic, renal, digestive, cerebral, skeletal muscle and myocardial tissue through paracrine and endocrine mechanisms (5, 7–10).

One hallmark feature in obesity is the ectopic and visceral adipose tissue (VAT) accumulation leading to AT transcriptome and secretome modification due to adipocyte hypertrophy and hyperplasia. This condition is related to tissue´s hypoxia and fibrosis, immune cell infiltration, stimulating the release of pro-inflammatory, pro-atherogenic and anti-angiogenic substances that affect AT biology and communication with other target tissues (11). In addition, myocardial cells are also affected by signaling molecules from the dysfunctional or “sick” AT (SickAT), given their link with heart hypertrophy and fibrosis, atrial fibrillation (AF), MI, among other CVD (12–15).

These data highlight the importance of establishing therapeutic tools to help combat obesity and, by extension, CVD. In a nutshell, obesity etiology is derived from an energy imbalance produced in the context of an obesogenic lifestyle (16) characterized by a hypercaloric diet and insufficient physical activity (PA) to counteract the SickAT expansion and subsequent defective signaling processes (10, 17, 18). Hence, PA and nutritional interventions (NI) might improve the SickAT profile and, consequently, enhance adipose tissue and myocardiocyte crosstalk. Therefore, this review discusses both AT and SickAT distribution and biology and their relationship with myocardial tissue. We will also address the molecular mechanisms by which exercise, food supplementation, and changes in eating habits can counteract obesity, taking as a pivotal point the role of the gut microbiota (GM) in SickAT pathogenesis to establish the non-pharmacological treatment of CVD.

## 2 The Sick Adipose Tissue: From Distribution to Interaction

The AT is a dynamic and anatomically heterogeneous organ acting as connective tissue throughout our organism. Beyond its particular vasculature, innervation and predominant adipocyte content, its microenvironment includes numerous immune cells, endothelial and stromal cells, fibroblasts, preadipocytes, and abundant extracellular matrix (ECM) (19–21). Each component possesses characteristic properties and can secrete various hormones, growth factors, microRNAs (miRNAs), cytokines, and chemokines coordinated, with autocrine, endocrine, and paracrine action on neighboring and remote organs/or cells (12, 22, 23). AT can also be classified by anatomical location, embryonic origin, morphology or function, the latter which can be grouped into white (WAT), brown adipose tissue (BAT) (24).

WAT is responsible for storing energy as fatty acids (FA) within triacylglycerides (TAG), supplying energy and controlling metabolic homeostasis through the white adipocyte endocrine functions (25). The main fat deposit in mammals is widely distributed throughout the subcutaneous adipose tissue (SCAT), gonadal and inguinal adipose depots. Adipose tissue located in the abdominal cavity, including intrahepatic and mesenteric, omental, and retroperitoneal fat, can be considered VAT (18, 19). Other intrathoracic AT depots identified include epicardial adipose tissue (EAT), occupying the space between the pericardium and myocardium, with a direct relationship with the coronary arteries; pericardial (PAT), located between the visceral and parietal pericardium, and perivascular (PVAT), which surrounds the remaining blood vessels (22, 26). It should be noted that both VAT and cardiovascular system (CVS)-based depots are considered a risk factor for cardiometabolic diseases, an association that has been widely reported (11, 26).

Unlike WAT, BAT has adipocytes with smaller lipid droplets, more abundant mitochondria and substantial vascularization, which provide its characteristic brown color (27). Likewise, BAT has high levels of uncoupling protein 1 (UCP1), which confer thermogenic properties by uncoupling between respiration and ATP synthesis during the FA oxidation in adipocytes (28, 29); hence, UCP1 is recently considered as a potential therapeutic target against obesity (30). In humans, BAT is found in specific areas (supraclavicular fossa, interscapular and paravertebral regions, in the axilla and nape) and represents only 4.3% of the total fat mass (31, 32). Notably, another type of adipocyte has been characterized within WAT deposits, and it has shown mixed characteristics of both white and brown adipocytes. For that reason, this new type of adipocyte has been coined as beige adipose tissue (BeAT). As stated above, BeAT reside within the WAT and can be mainly found within the inguinal WAT (33). Also, BeAT express the UCP1 gene and, by extension, thermogenic properties (34). Note that this browning process occurs through exposure to cold, β-adrenergic stimulation and pharmacological modulation of WAT (35).

### 2.1 Changes in Adipose Tissue Microenvironment and Meta-Inflammation: The Sick Forgotten

According to the WHO, obesity is defined as excessive or abnormal fat accumulation with negative health repercussions, determined by a body mass index (BMI) ≥ of 30 kg/m^2^ (36). Although its etiology includes genetic, social, environmental and/or cultural factors, in most cases, it is characterized by an imbalance between energy intake and energy expenditure, attributed to poor eating habits and sedentary lifestyles (16). This hypercaloric or overnourished state leads to more significant fat accumulation in AT, mainly in the form of ectopic or visceral depots (37). AT can increase in abundance through two different processes: hypertrophy and hyperplasia or new adipocytes formation.

**Figure 1:**
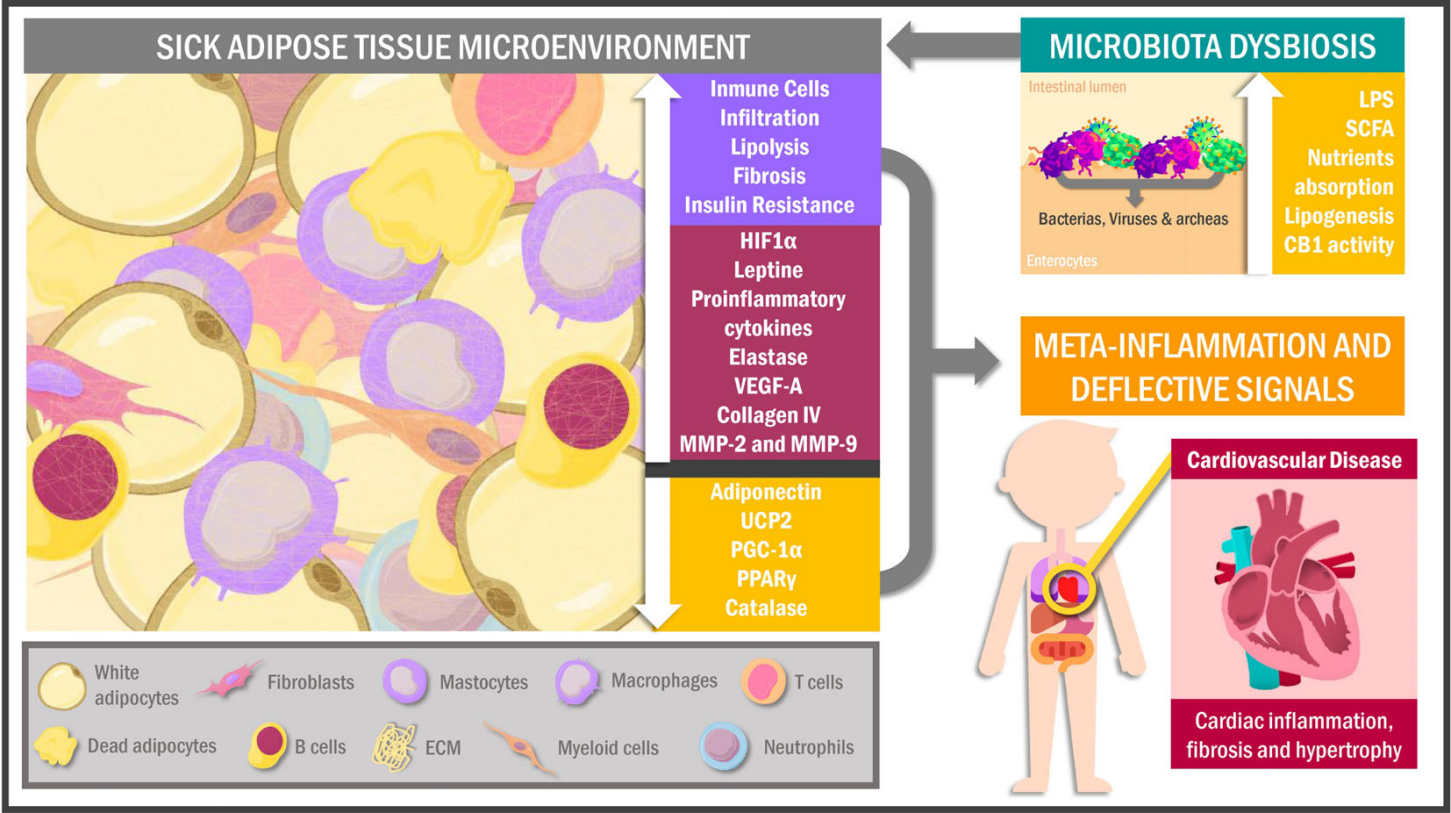
Sick adipose tissue microenvironment and its interactions. Hypertrophic adipocytes and immune cells infiltration characterize the adipose tissue of obese individuals in response to a hypoxic environment as a signal for cell death and inflammation. This phenomenon leads to proteomic dysregulation and deflective peripheral signals promoting metabolic alterations in other tissues like muscle cells, particularly the myocardiocytes. In addition, obesogenic habits in overweighed people cause changes in the intestinal microbiota triggering adipose tissue chronic inflammation and cellular senescence. UCP2, uncoupling protein 2; PGC-1α, peroxisome proliferator-activated receptor gamma coactivator 1α; PPARγ, peroxisome proliferator-activated receptor gamma; VEGF-A, vascular endothelial growth factor A; MMP, metalloproteinases; HIF1α, hypoxia-inducible factor 1α; LPS, lipopolysaccharides; SCFA, short-chain fatty acids; CB1, cannabinoid receptor 1; ECM, extracellular matrix.

Hyperplasia is considered a beneficial and adaptive process by which new functional adipocytes can be formed from fibroblastic preadipocytes without altering their secretory profile and maintaining vascularization of the AT microenvironment (37, 38), which is associated with better metabolic health (39). A transcriptional cascade regulates this cell line differentiation carried out by peroxisome proliferator-activated receptor gamma (PPAR *γ*) and CCAAT enhancer-binding proteins (C/EBP), in conjunction with pro-adipogenic factors such as bone morphogenetic proteins (BMPs) (40, 41). However, hypertrophy and subsequent adipocyte dysfunction disrupt these signaling processes and preserve the pro-inflammatory phenotype characteristic of obese individuals (**Figure 1**) (42, 43).

The pre-existing adipocytes gain volume *via* increased fat accumulation, experiencing heightened mechanical stress by contact with adjacent cells and other extracellular matrix components (ECM) (44). Over time, AT expansion results in reduced regional blood flow, altered oxygen diffusion and finally tissue hypoxia, all of that related to both oxidative stress activation (OS) (45) and increased transcriptional activity of hypoxia-inducible factor 1α (HIF1α), nuclear factor kappa B (NF-kB), and cAMP response element-binding protein (CREB) genes, whose transcripts, in turn, drives to adipokines, chemokines, metalloproteases and growth factors gene expression, all of these related to a pro-inflammatory peptidic secretome (46). Concurrently, these hypoxia-induced factors downregulate anti-inflammatory and metabolism-regulatory adipokines such as adiponectin, which occurs alongside reduced transcription of antioxidant and thermoregulation-related genes, particularly catalase encoders UCP2, PPARγ and peroxisome proliferator-activated receptor-gamma coactivator 1α (PGC-1α) (47–49). Consequently, transcriptomic and proteomic changes in AT lead to a low-grade inflammatory environment characterized by functionally-altered fibroblasts, endothelial cells and immune cells niche (50, 51). Regarding the latter, macrophages have been identified as the predominant cells of this system in AT, showing a pro-inflammatory M1 phenotype in obese individuals compared to the anti-inflammatory M2 in lean individuals (52). In this scenario, the hypoxic inflammatory state of AT promotes the release of interferon-γ (IF-γ) by T helper 1 (Th1) lymphocytes, inducing M1 macrophage recruitment and polarization, which causes increased release of pro-inflammatory cytokines such as tumor necrosis factor-alpha (TNF-α), monocyte chemoattractant protein 1 (MCP-1), interleukin (IL) -6, IL-12, IL-1β and IL-23 (53–56).

Obese patients exhibit clusters of lipid-binding macrophages from dead adipocytes, a phenomenon well-correlated with AT inflammation and insulin resistance (57, 58). In addition, obesity promotes CD8 + and CD4 + T lymphocytes infiltration together with effector B-cells, heightening pro-inflammatory factors release and consequently AT dysfunction with defective extracellular signaling (59, 60). Similarly, myeloid cells, mast cells (50, 51) and neutrophils are also present in SickAT, showing that they contribute to tissue damage through elastase secretion and thus promote macrophage recruitment (61, 62).

Among the essential SickAT characteristics in obese patients are altered angiogenesis and endothelial dysfunction (ED). Although SickAT upregulates vascular endothelial growth factor A (VEGF-A) and HIF1α expression (both linked to angiogenesis), production is insufficient to generate neovascularization and counteract hypoxia, inflammation and necrosis characteristic of obese patients (63–66). Furthermore, SickAT leads to reactivity in the endothelium of the surrounding vessels, inducing the synthesis of intracellular adhesion molecule (ICAM-1), P-selectin and E-selectin, which in turn promotes macrophage infiltration worsening the pro-inflammatory milieu (67). Additionally, adipocyte-endothelial crosstalk can contribute to vasomotor alterations, deteriorating the oxygen bioavailability in EAT, PVAT and PAT (68, 69).

Likewise, HIF1α upregulation, immune cell infiltration and hyperactivity are associated with AT fibrosis. Remarkably, the increased synthesis of ECM components, mainly type-VI collagen and its cleavage products such as endotrophin, have been associated with metabolic dysfunction in obese mice *via* mechanical stress caused by limits on AT expansion (44, 70–72). Interestingly, HIF1α expression is correlated with metalloproteinases (MMP) -2 and MMP-9 in EAT, which are considered necessary for expansion and secretome alterations (69).

On a different plane, adipocyte metabolic activity is substantially modified in a hypoxic state. In fact, some glycolytic enzyme genes such as hexokinase 2 (HK2), phosphofructokinase (PFKP) and GLUT1 exhibit an increased expression in adipocyte cell cultures under hypoxic conditions (73, 74). Furthermore, although GLUT4 is the main isoform found in adipocytes, GLUT1 is the most efficient glucose transporter at low-oxygen levels (75). As expected in hypoxic states, the above changes suggest adipocytes have increased glucose uptake and metabolism (76), as confirmed by their increased lactate secretion (77).

In summary, lipid metabolism proteomics tends towards lipolytic extreme under hypoxic conditions (78). The SickAT microenvironment is characterized by multiple agents influencing insulin signaling, like IL-6, TNF-α, resistin, and IL-1β (79). Under normal conditions, insulin inhibits lipolysis through the mTORC1-Egr1-ATGL pathway, so inhibition of insulin´s second messengers cascade increases lipolytic activity (80). Furthermore, fatty acid uptake by adipocytes is blunted under hypoxic conditions (74), leading to plasma free fatty acids increase worsening insulin signaling (81) and contributing to the pro-inflammatory state (82). It should be highlighted that intrathoracic and visceral AT, BAT, BeAT and SCAT depots are affected in obesity (83), the thermogenic properties of BAT can be disturbed by mild inflammatory cells infiltration in severely obese individuals (84), leading to diminished glucose and FFA oxidative metabolism, and therefore contribute to IR and dyslipidemia development (84–86). In contrast, BeAT occurs less frequently owing to the dysfunctional state of WAT in obese patients (87).

### 2.2 Microbiota Dysbiosis

The gastrointestinal tract contains a complex population of microorganisms, the gut microbiota (GM), which exerts a marked influence on human health and disease (88). Multiple factors contribute to establishing the intestinal microbiota during early childhood and as it evolves into adulthood, but it is not hard to imagine that one of the main factors that shape the gut microbiota structure throughout our lives is our diet. In addition, gut bacteria play a crucial role in maintaining and proper function of the immune system and intermediary metabolism. Abnormalities in the intestinal bacterial composition (dysbiosis) have been associated with many inflammatory, infectious, autoimmune and metabolic diseases.

GM is constituted by bacteria, *archaea*, viruses and fungi, interacting symbiotically with the host (88). However, hypercaloric diet (HCD) and obesogenic habits alter the microbiota-host relationship, affecting its composition and interaction with the organism (89). A growing body of evidence in this area has centered on comparing energy and body fat storage in germ-free mice with transplanted microbiota of wild mice or obese individuals. The findings were that although the mice maintained the same diet in both cases, there was a substantial increase in adiposity and IR development after microbial transplantation, which could be attributed to the role of the microbiota in calorie extraction and absorption (90–92). Although the possible mechanisms triggered by HCD and obesity involved in the GM-AT axis interaction have not been fully elucidated yet, specific hypotheses have been proposed to explain these findings.

Significant among these theories is the influence of microbial products on AT. In physiological situations, the intestinal wall has selective permeability due to the tight junction proteins between enterocytes; however, an HCD can decrease expression of these proteins and allow passage of lipopolysaccharides (LPS), bacterial products of gram-negative bacteria (93–95). Once in circulation, LPS spread throughout the body and act on type 4 toll receptors (TLRs) located in AT adipocytes and immune cells (96), activating pathways dependent on myeloid differentiation factor 88- (MyD88-) and TIR-domain-containing adapter-inducing interferon-β (TRIF). This process activates the nuclear translocation of NF-kB and the subsequent release of pro-inflammatory substances, contributing to the typic low-grade inflammation seen in SickAT (97, 98). Furthermore, it has been reported that LPS/TLR4 pathway activation can decrease WAT browning (99) and adaptive thermogenesis (100). Another interesting observation is that GD can increase permeability by activating the intestinal endocannabinoid system, acting on its CB1 receptors associated with obesogenic habits (101).

Likewise, several commensal bacteria species of the GM ferment indigestible carbohydrates and fiber to obtain energy by forming short-chain fatty acids (SCFA) (102–104), mainly acetate, butyrate and propionate. These metabolites have key roles in energy metabolism (105) and immunomodulation (106), by acting on the family of free fatty acid receptors (FFAR), especially FFAR2 (GPR43) and FFAR3 (GPR41), located in gastrointestinal, nervous, and AT tissue (107). Therefore, GD present in obese individuals may lead to changes in SCFA levels and, by extension, SickAT-related metabolic alterations. Higher SCFA production has been reported to promote lipogenesis by activating carbohydrate responsive element-binding protein (ChREBP) and sterol regulatory element-binding transcription factor 1 (SREBP1), favoring weight gain in animals models (108, 109). Similarly, studies have shown that SCFAs can inhibit fasting-induced adipocyte factor (FIAF), which can suppress enzyme lipoprotein lipase (LPL) activity and thus increase triacylglycerol (TAG) storage and accumulation in AT (90, 91).

Additionally, SCFAs stimulates peptide YY (PYY) and glucagon-like peptide 1 (GLP-1) secretion, which in turn slow down the intestinal transit time and thus increase nutrient absorption (109, 110), influencing appetite control (111). Other GT-AT axis-related mechanisms such as the TMA/FMO3/TMAO signaling pathway (112), nucleotide-binding oligomerization domain-containing 1 (NOD1) and NOD2 (113) proteins, and modulation of the miRNA-181 family (114) have also been explored in the context of obesity and its possible implications in the switch to SickAT. However, given the lack of a proven causal link between microorganisms, their products and specific mechanisms in humans, together with the heterogeneity of GT and the fact that *Bacteroidetes* and *Firmicutes* are predominant in both obese and healthy individuals (115), further research is warranted in this area.

## 3 Intercellular Signaling Between Adipocytes and Myocardial Cells

### 3.1 Adipokines

#### 3.1.1 Leptin

Leptin is a peptidic hormone secreted by AT, so peripheral leptin levels tend to remain directly proportional to AT volume (116). Consistent with this finding, obese patients show elevated leptin levels, but signaling defects mean that appetite suppression is reduced or nullified (117). Thus, obesity-related hyperleptinemia has been suggested as an important factor in CVD genesis (118). From a molecular perspective, leptin plays a role in atherosclerosis initiation by the hyper-production of reactive oxygen species (ROS) in endothelial cells (119). The explanation of this phenomenon relies on increased fatty acid oxidation *via* protein kinase A stimulation, which increases MCP-1 production, facilitating macrophage infiltration into the sub-endothelial (120). Furthermore, *in vitro* studies have shown that leptin increases cholesterol uptake in macrophages by ACAT1 modulation (121). These results match with clinical findings obtained in other studies; indeed, leptin levels are correlated with markers of atherosclerosis such as the intima-media thickness of the carotid artery (122) and likewise with the severity of coronary artery disease (CAD) (123).

It has also been hypothesized that leptin can induce cardiomyocyte hypertrophy (124). This effect seems mediated by multiple mechanisms, such as increased endothelin 1 (ET-1) and ROS production in cardiomyocytes in response to leptin levels (125). Another theory is that leptin activates the mTOR (126) and PPAR-α signaling pathways (127). Consistent with the above, clinical studies have shown a positive correlation between serum leptin levels and left ventricular thickness in obese or insulin-resistant patients (128). In contrast, another study conducted in a murine model proposes that leptin exhibits antihypertrophic properties. Based on these findings, mice with left ventricular hypertrophy reverted to normal ventricular function when normal leptin levels were restored (129).

Nonetheless, rather than a direct consequence of restored leptin levels, these findings may stem from reversing metabolic alterations inherent to leptin deficiency, so these results should be interpreted cautiously. On the other hand, the antihypertrophic properties associated with leptin levels have been reported in some studies (130–132). In conclusion, it remains uncertain whether cardiac hypertrophy is due to leptin pro-hypertrophic action or is instead an effect of resistance to leptin antihypertrophic action on cardiac remodeling.

#### 3.1.2 Interleukin 6

As AT produces around a third of circulating IL-6, it can be considered an adipokine (133); however, its role in cardiomyocyte function is somewhat controversial. In acute phases, IL-6 signaling has been attributed a cardioprotective effect by inducing anti-apoptotic pathways and conferring protection against OS (134). However, IL-6 also decreases myocardial contractility and eventually increases nitric oxide (NO) production may be through inducible nitric oxide synthase (iNOS) activation (135, 136). Likewise, a study in animals reported no significant effects of treatment with IL-6 on left ventricular remodeling (137), while another study found that IL-6 signaling blockade suppresses myocardial inflammation and ventricular remodeling (137). Since human and murine IL-6 show only 41% similarity, animal studies should be approached with caution. Regarding human studies, elevated IL-6 levels have been correlated with ventricular dysfunction (138), heart failure, arrhythmias and worse clinical outcomes (139), indicating a need for further study to clarify the role of IL-6 in CVD.

#### 3.1.3 Adiponectin

Under SickAT conditions, adiponectin secretion is considerably reduced, impacting negatively on cardiovascular function (140). On the other hand, normal adiponectin levels have been shown to improve cardiomyocyte dysfunction in animal models, probably due to mechanisms related to IRS-1 and the c-Jun pathway (141). Furthermore, adiponectin is necessary to activate PPARγ signaling, which confers protection against myocardial hypertrophy and cardiac remodeling (142). Likewise, adiponectin inhibits iNOS and NADPH oxidase expression, decreasing OS under ischemic conditions (143). On a different level, adiponectin stimulates COX-2 expression and prostaglandin E2 synthesis, conferring cardioprotective and anti-inflammatory properties (144).

Clinically, hypoadiponectinemia is independently associated with ED (145), while normal adiponectin levels are associated with a lower risk of ischemic events in men (146). Conversely, low adiponectin levels positively correlate with left ventricular hypertrophy, regardless of age or other metabolic factors (147). However, a systematic review found no significant relationship between adiponectin levels and cardiovascular mortality, and a 10% increased risk of death from any cause was reported (148). This finding requires considering concurrent situations such as kidney failure and age-related adiponectin resistance, leading to bias when analyzing different populations (149, 150). Nonetheless, the prevailing view in the literature is that adiponectin confers cardioprotection at normal concentrations, while hypoadiponectinemia is related to an increased risk of developing ED as well as myocardial dysfunction.

### 3.2 BCAAs

Branched-chain amino acids (BCAAs), valine, leucine and isoleucine, are essential amino acids playing a critical energetic role in different tissues, including myocardial cells and adipocytes (151). For example, in physiological circumstances, adipocytes oxidize BCAAs as an important energy substrate; however, different stimuli or organic conditions such as insulin resistance, obesity or cardiovascular disease cause adipose cells reprogramming, reducing BCAA metabolism in the heart, AT and liver (152–154).

**Figure 2:**
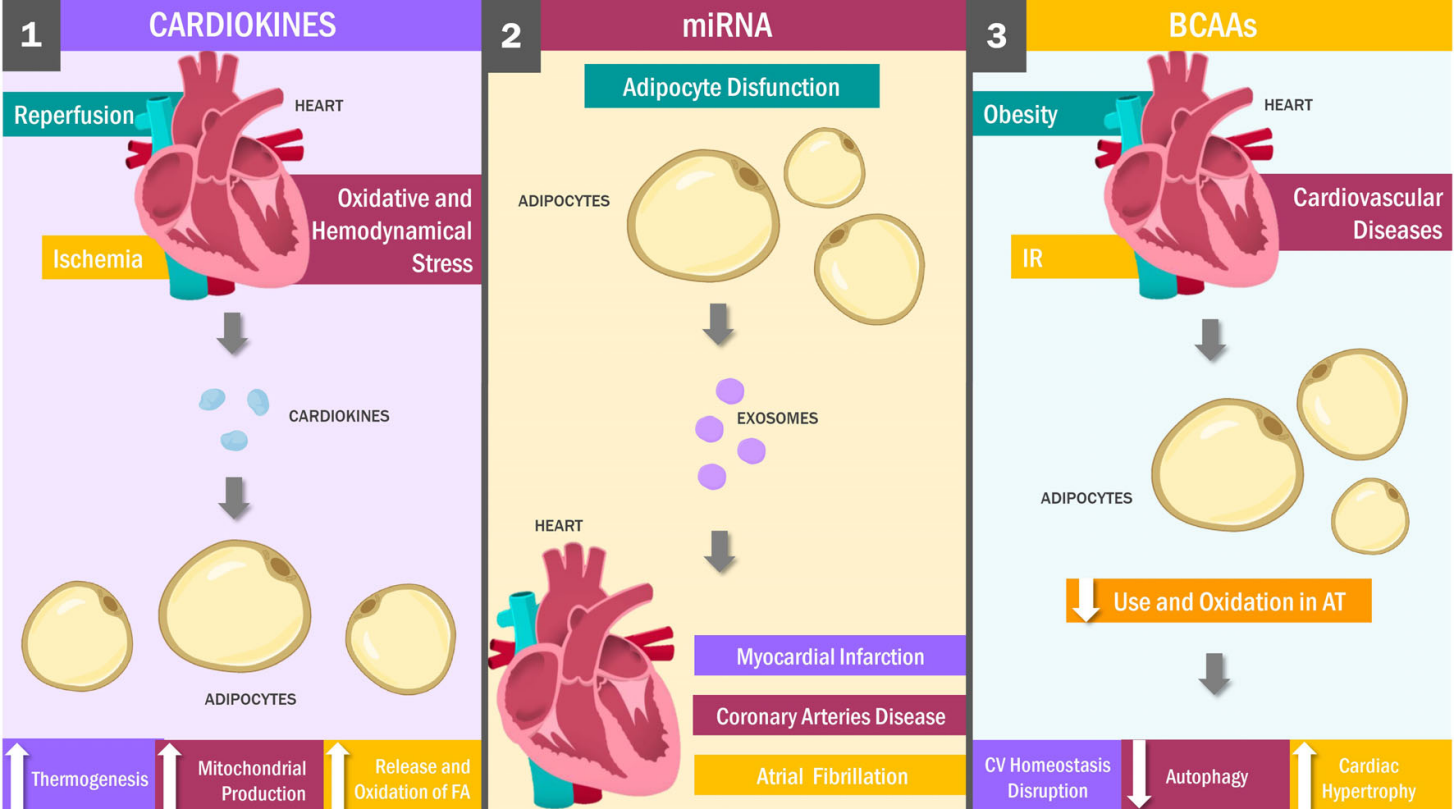
Heart and Adipose Tissue Crosstalk: Key Messengers. Cardiokines: stimuli such as cardiac ischemia, reperfusion, oxidative and hemodynamic stress stimulate the production of cardiokines, which signaling in an endocrine and paracrine mechanism to the adipose tissue promoting weight loss by increasing thermogenesis and both the release and oxidation of fatty acids. Adipose tissue dysfunction is a stimulus for miRNAs release, which travel through the bloodstream to the myocardial tissue inside exosomes, exerting cardioprotective against myocardial infarction, coronary artery disease and atrial fibrillation. On the other hand, obesity, IR and cardiovascular diseases decrease BCAAs oxidation in adipose tissue, which decreases autophagy with heart hypertrophy and, finally, the alteration of the bioenergetic homeostasis of the heart. FA, Fatty Acids; AT, Adipose Tissue; CV, Cardiovascular; IR, Insulin resistance.

The mechanisms underlying these changes have not been fully elucidated yet; however, epigenetic changes such as PP2Cm, KLF15, or GRK2 gene expression during heart disease could modify the cells’ metabolic profile. Subsequently, alterations in BCAA catabolism and use caused by these metabolic changes could lead to rising arterial amino acid levels (27, 153). Likewise, AT inflammation has been linked to tricarboxylic acid cycle modifications, resulting in reduced BCAA catabolism and use, which provides an alternative explanation for the accumulation of amino acids in plasma (155, 156) (**Figure 2**). These variations in local and organic BCAA concentrations lead to chronic mTOR receptor expression in myocardial cells, and thus, autophagy suppression pathways induction, alterations in insulin sensitivity and tissue transport, as well as protein synthesis pathway activation, promoting the inhibition of autophagy protective functions, by modifying the bioenergetic heart homeostasis and cardiac hypertrophy stimulation, respectively (157).

Given these findings, it is not surprising that a correlation between heart failure and elevated BCAA levels has been found in numerous studies (152, 158). For example, a clinical trial conducted by Peterson et al. evaluated total amino acid concentrations in patients with heart failure, finding them to be abnormally high (159). Similarly, results reported by Kato et al. indicated elevated plasma amino acid levels as a consequence of metabolic changes in sodium-sensitive hypertensive rodents (160). In contrast, a clinical trial conducted by Aquilani et al. reports a decreased BCAA levels in patients with chronic heart failure compared to healthy individuals. Although these results could seem contradictory, factors such as the site of amino acid quantification and the variability in BCAA levels due to both duration and severity of pre-existing disease could explain the differences between findings (161). In this regard, AT and cardiac tissue exert a reciprocal influence on each other in various pathological scenarios *via* modifications in BCAA catabolism and consumption (154–156, 162).

### 3.3 Cardiokines

The heart is conventionally viewed as a contractile organ acting as a muscular pump to provide nutrients to the body (163). Beyond these functions, however, it can exert regulatory actions on other organs, such as the kidney, liver or AT (164). These modulatory activities are carried out mainly through molecules synthesized and secreted by the heart, known as cardiokines (165–168).

To date, it has identified up to 16 cardiokines, which are thought to exert homeostatic functions related to growth, cell death, fibrosis, hypertrophy and cardiac remodeling. In addition, although these molecules have predominantly paracrine and autocrine functions, certain cardiokines show endocrine mechanisms of action, allowing them to act on distant tissues (169). Such is the case of the firsts cardiokines identified, known as atrial (ANP) and brain (BNP) natriuretic peptides (NPs) (164, 170). Besides their participation as blood pressure regulators, both peptides play a critical role in modulating AT energy metabolism (171).

In this way, different stimuli such as ischemia, reperfusion, OS, hemodynamic stress, and cardiac hypertrophy can trigger NP synthesis and release by different cardiac cells into the circulation and ultimately reaching the AT (172). In this tissue, NPs bind to the NPR-A receptors, activating the guanylyl cyclase and cGMP formation. This process, in turn, activates the PKG, an enzyme responsible for phosphorylating key factors such as UCP-1, PPARGC1A, CYCS, PRD1-BF 1 and RIZ1, inducing white adipocyte browning, increasing lipogenesis, mitochondrial biogenesis and lipid oxidation (171, 173).

Collectively, these phenomena have a double effect. Fatty acids are released into the bloodstream as energy substrates to compensate for the low heart contractility observed during the abovementioned pathological scenarios (174), while increased mitochondrial production, thermogenesis, and fatty acid oxidation promote weight loss (175, 176). These reports were verified by other studies showing abnormally elevated NP concentrations during CVD and decreased levels of these peptides in obese individuals (164, 177). For example, in one study carried out by Kovacova et al. (29), the NPRR expression was significantly lower in obese than normal-weight individuals. These findings replicated those obtained in studies carried out in humans and murine, wherein plasma and cardiac levels of both BNPs and ANPs were significantly lower in obese than normal-weight subjects (175, 178).

### 3.4 miRNAs and EVs

miRNAs are small, non-coding RNA molecules functioning as regulatory agents in numerous physiological and pathological processes by participating in post-transcriptional mRNA and translation into protein processes (179, 180). These molecules are synthesized in response to a wide range of stimuli by different tissues (181), among which AT and EAT are responsible for the production and release of multiple miRNA varieties (14, 182).

Most miRNAs on EAT operate through autocrine fashion and have been implicated in various AT processes such as adipocyte differentiation, fatty acid metabolism, cholesterol homeostasis, adipogenesis, browning and inflammation (183–185). Other miRNAs are released into the circulation *via* exosomes, from where they travel to and penetrate the heart or other distant organs (186). Although it has been established that EAT releases different miRNAs towards the heart in response to tissue dysfunction or certain specific stimuli (13), but the functions and underlying mechanisms of action have not been fully characterized. Nonetheless, recent studies have identified new miRNAs and their potential role in the pathogenesis and development of heart diseases (12, 187, 188).

In this vein, miRNAs have been implicated in atrial fibrillation (AF), as demonstrated in the study carried out by Liu et al. (189), wherein miR-320d were transported *in vitro* by exosomes to FA cardiomyocytes, revealing enhanced cell viability and decreased post-transfection cardiomyocytes apoptosis, reversing several FA characteristic effects by inhibiting factor STAT3. Likewise, a possible cardioprotective role has been suggested to miR-146a due to an inhibitory effect on early growth response factor-1 (EGRF1) in suppressing typical post-MI phenomena such as apoptosis, inflammatory responses and cardiac fibrosis (190). Similar results were obtained by Luo et al., in which miR-126 overexpression in hypoxic H9c2 cells led to reduced local inflammation, pro-fibrotic protein expression, and microvasculature and cell migration, thus mitigating the effects of cardiac injury in the infarcted area (191).

Numerous miRNAs play a positive role in some cardiac pathologies beyond acute myocardial infarction (AMI) and AF, including CAD. For example, it has been shown that during CAD progression, miRNA-3614 expression is downregulated in EAT, which produces an inhibitory effect on factors such as TRAF6, which regulates immune cell recruitment and activation as apoptosis and cardiac remodeling during myocardial ischemia (189, 192). In this context, a study by Zou et al. identified miR-410-5p and its promoting effects on cardiac fibrosis in mice with regular diets by silencing Smad7; concurrently, miR-410-5p demonstrated anti-fibrotic effects in mice fed high-fat diets (193). These results suggest a dual role for miRNAs in cardiovascular pathologies; besides the cardioprotective role of some miRNAs, these molecules can exert harmful effects on cardiac tissue, promoting effects such as local inflammation, hypertrophy, remodeling and cardiac fibrosis in different CVDs (183, 194–198).

## 4 Non-Pharmacological Approach to Adipocyte-Myocardiocyte Defective Signaling: Impact of Lifestyle

Preclinical and clinical evidence suggests that positive lifestyle changes derived from increased PA and NI could improve the above-described pro-inflammatory metabolic status of obese patients, highlighting their utility as possible non-pharmacological therapeutic strategies to manage obesity and cardiovascular risk.

In this regard, studies suggest that PA reduces circulating levels of insulin, leptin, and pro-inflammatory cytokines and raise adiponectin and apelin concentrations (199–202). In addition, increasing PA has been linked to heightened endothelial NOS (eNOS) expression and iNOS expression reduction (199, 203). These findings suggest that PA as a strategy helps restore a healthy metabolic state at the preclinical level.

Additionally, clinical studies have reported an anti-inflammatory, cardioprotective and slimming effect of PA. For example, a study in obese men showed that exercise was more effective than diet in reducing body weight (BW), improving the systemic inflammatory profile and IR and circulating levels of adipokines (204). Likewise, a study conducted in obese patients with T2DM subjected to dietary restriction and aerobic exercise reported that after a 3-month intervention, adiponectin levels rose while BMI and TNF-α, IL-6 and leptin levels fell significantly (205).

Concerning the different intensities of PA, a clinical trial demonstrated that moderate exercise combined with calorie restriction aided in normalizing adiponectin, leptin and resistin levels in obese adolescents (206). Furthermore, a meta-analysis performed by Maillard et al. (207) reported that high-intensity interval training effectively reduced SCAT and VAT. Similarly, another meta-analysis found that both moderate and high-intensity PA have a similar effect on weight reduction and body composition; however, results were seen more quickly when performing high-intensity exercise (208). Therefore, besides its anti-inflammatory properties, exercise can reduce BW, indirectly counteracting SickAT defective signaling by modifying its composition.

Studies have also demonstrated that PA has a regulatory effect on circulating microRNAs in individuals with cardiometabolic abnormalities. In this context, a clinical trial showed that circulating levels of miR-192 and miR-193b (associated with a prediabetic state) were modified after 16-week exercise intervention (209). Along similar lines, a combined aerobic and resistance exercise program in obese patients for three months was associated with significantly decreased levels of the inflammatory miRNA miR-146a-5p (210).

Aside from the weight loss achieved with exercise, dietary interventions have also been shown to positively impact AT and CVS crosstalk. In this regard, it has been proven that caloric restriction in the rat diet causes significantly reduced expression of iNOS, TNF-α and IL-1β in PVAT (211). Furthermore, another study conducted in rats showed that calorie control-induced weight loss was associated with improved endothelial NOS function, reduced TNF-α levels and normalized plasma adipokines y hormones levels such as leptin and insulin (212). Therefore, diet is a rationale tool to improve the cardiovascular functionality of the PVAT.

In another study, Kim et al. showed that intermittent fasting (IF) with an isocaloric diet increased VEGF expression in WAT, favoring macrophage polarization towards the M2 phenotype, which is linked to increased thermogenesis and AT browning (213). In this regard, a clinical trial in obese patients reported that IF combined with caloric restriction and liquid meals promotes significant BW loss and improves risk indicators for CAD (214). Furthermore, other studies conducted by the same research group (215) and Trepanowski et al. (216) were able to show that in addition to reducing BW, the abovementioned diet decreases levels of leptin IL-6, TNF-α and insulin-like growth factor-1 (IGF-1). These results point to IF and low-calorie diets as a possible strategy to manage AT visceral adiposity and secretory profile, owing to their cardioprotective effect.

Regarding the role of the nutritional maneuvers approach on circulating microRNAs, Hsieh et al. (217) showed through a preclinical study that a low-fat diet could reverse obesity-associated inflammatory miRNA profiles *via* BW reduction. Consistent with this finding, evidence in humans suggests that BW loss achieved by very-low-calorie NI in obese women (218) or protein-rich diets in obese men (219) allow positive modulation of circulating levels of different miRNAs such as miR-34a, miR-208, miR-193a, miR-223, miR-320, miR-433, miR-568 and miR-181a.

Likewise, preclinical and clinical studies have shown the prebiotic and probiotic effects in reducing cardiovascular risk by leptin resistance (220) and leptin level reductions (221, 222). In addition, an adiponectin increase (223, 224) and lowering both apelin (225) and ANP levels (226) have been consistently reported, a fact attributed to HCD-induced GD correction and thus a reduced LPS-induced endotoxemia and SCFA levels. Likewise, 3-n PUFA supplementation has been associated with recovery of the adipokine and cardiokine profile, resulting in a healthier cardio-metabolic state. In this context, studies in animal models and humans have linked supplements administration with a significant reduction in leptin (227, 228), follistatin-like 1 (229) and BNP levels (230), and adiponectin increase (231). Finally, polyphenols such as lycopene, resveratrol and curcumin have also been linked to improved inflammatory and adipokine profile, body composition and cardiac fibrosis/hypertrophy in study subjects (232–235).

These data suggest that PA and different NI, either alone or in combination, are associated with the upregulation of adipokines, cardiokines, miRNAs and other components associated with crosstalk between AT and CVS. Therefore, these strategies are beneficial in reducing cardiovascular risk in obese patients due to their mechanisms capable of counteracting the characteristic pro-inflammatory state of SickAT.

## 5 Conclusions

Adipose tissue is a multifunctional exhibiting well-characterized inter-organ paracrine and endocrine networking, including myocardial tissue communication. Obesity is characterized by metabolic changes in SickAT caused by a hypoxic microenvironment due to adipocyte hypertrophy driving to immune cell infiltration and a systemic pro-inflammatory state affecting target cells such as cardiomyocytes. Excessive adipokines, microRNA, BCAAs characterize SickAT defective signaling, and other pro-inflammatory substances release altering myocardial cells function and, consequently, CVD development. Likewise, heart cells can also alter AT signals, thereby causing a vicious cycle that fuels meta-inflammation. Under this premise, lifestyle changes such as PA, low-calorie diets, IF, and food supplementation are fundamental non-pharmacological therapeutic tools to combat obesity and CVD due to their identified regulatory mechanisms in AT and CVS signaling.

## Author Contributions

Conceptualization: PD, MN, CC, and VB. Investigation: LD’M, MD, RC, MN, and MC. Writing – original draft: PD, MD, RC, MC, MB, JG, and ER. Writing – review and editing: VB, CC, JR, MB, and LD’M, JG. Funding acquisition: VB and JG. All authors contributed to the article and approved the submitted version.

## Funding

This work was supported by research grant no. CC-0437-10-21-09-10 from Consejo de Desarrollo Científico, Humanístico y Tecnológico (CONDES), University of Zulia, and the research grant no. FZ-0058-2007 from Fundacite-Zulia.

## Conflict of Interest

The authors declare that the research was conducted in the absence of any commercial or financial relationships that could be construed as a potential conflict of interest.

## Publisher’s Note

All claims expressed in this article are solely those of the authors and do not necessarily represent those of their affiliated organizations, or those of the publisher, the editors and the reviewers. Any product that may be evaluated in this article, or claim that may be made by its manufacturer, is not guaranteed or endorsed by the publisher.

## Abbreviations

AF, atrial fibrillation; AMI, acute myocardial infarction; AT, adipose tissue; BAT, brown adipose tissue; BeAT, beige or brite adipose tissue; BMI, body mass index; BCAAs, Branched-chain amino acids; BW body weight; C/EBP, CCAAT-enhancer-binding proteins; CKD, chronic kidney disease; CVD, cardiovascular disease; CVS, cardiovascular system; EAT, epicardial adipose tissue; eNOS, endothelial nitric oxide synthase; FFA, free fatty acid; GD, gut dysbiosis; GM, gut microbiota; HCD, hypercaloric diet; IF, intermittent fasting; iNOS, inducible nitric oxide synthase; IR, insulin resistance; LPS, lipopolysaccharides; MetS, metabolic syndrome; NAFLD, non-alcoholic fatty liver disease; NI, nutritional intervention; NP, natriuretic peptide; OS, oxidative stress; PA, physical activity; PAT, pericardial adipose tissue; PPARγ, peroxisome proliferator activated receptor γ; PVAT, perivascular adipose tissue; SickAT, sick (dysfunctional) adipose tissue; SCAT, subcutaneous adipose tissue; SCFA, short-chain fatty acids; T2DM, type 2 diabetes mellitus; WAT, white adipose tissue; VAT, visceral adipose tissue.
